# Supplementary material for: Preselection of potential target spaces based on partial information by the supplementary eye field
Source: Commun Biol. 2024 Oct 4;7:1215. doi: 10.1038/s42003-024-06878-z (PMC11452695; doi:10.1038/s42003-024-06878-z)
Supplement: Supplementary file 2 — Supplementary Information [file 42003_2024_6878_MOESM2_ESM.pdf]

Supplementary information for

Preselection of potential target spaces based on partial information by the supplementary eye field

Osamu Yokoyama*, Yukio Nishimura

* Correspondence: Osamu Yokoyama, Ph.D.

Email: yokoyama-os@igakuken.or.jp

This PDF file includes:

Supplementary Figures 1 to 3

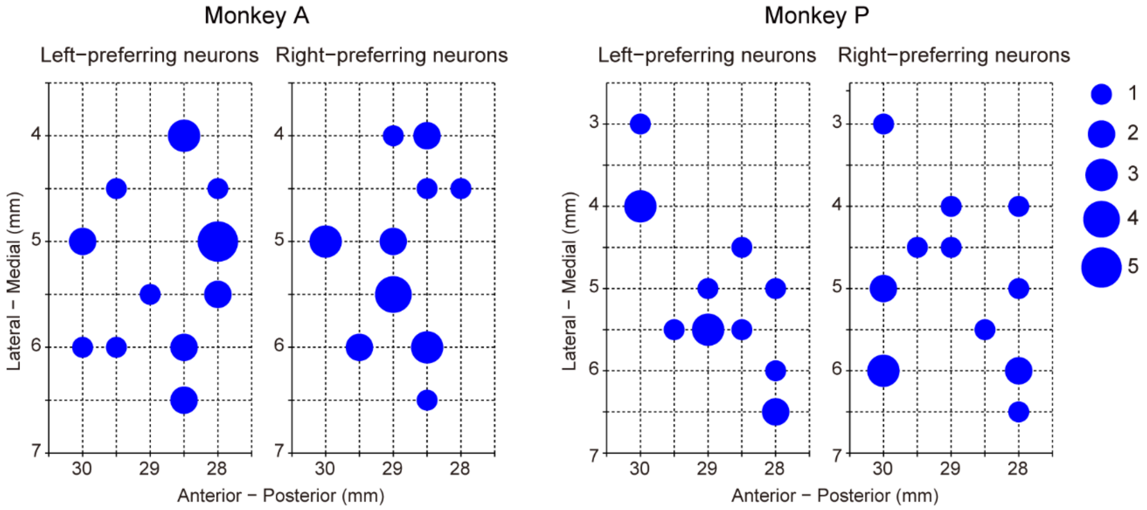

Supplementary Fig. 1. Spatial distribution of SEF neurons representing and preferring the left or right visual hemifield. Left/right-preferring neurons are neurons showing more spike counts in left/right-instructed trials than in right/left-instructed trials. The preference during 100–300 ms after the symbolic cue onset was examined for neurons that were judged to represent information about the potential target space (visual hemifield) during this period. The size of the circle indicates the number of neurons found along the depth of the electrode at that location.

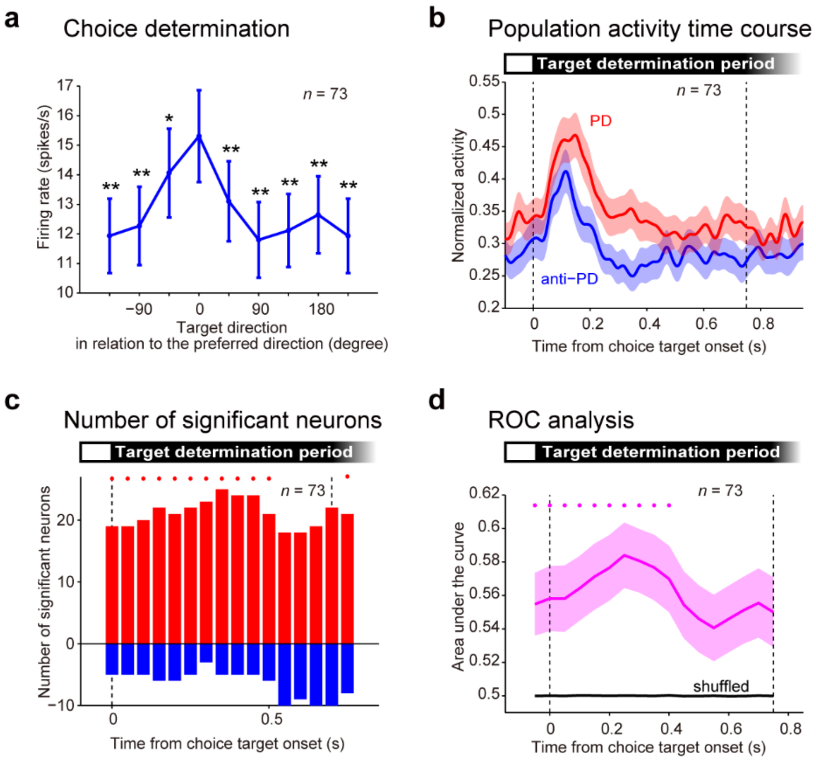

Supplementary Fig. 2. SEF neurons encode target position during the target determination period. a Population tuning curve at 0–200 ms after choice target onset. The PD was aligned across neurons at 0°. The error bar indicates SEM. Asterisk indicates that activity in that direction was less than that in the PD (paired *t* test, *p < 0.01, **p < 0.001). **b** Normalized population activity of neurons for which a PD could be determined (mean ± SEM). **c** The number of neurons that exhibited significantly different activity between the PD and anti-PD conditions as a function of time is displayed in the same format as in Fig. 4d. **d** Time course of the mean (±SEM) AUC values for the PD vs. anti-PD conditions across the neurons displayed in the same format as in Fig. 4e.

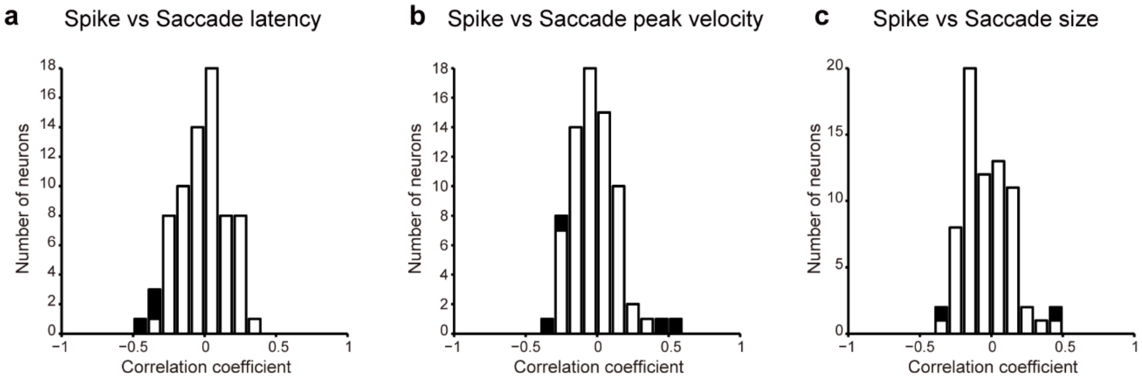

Supplementary Fig. 3. Distribution of trial-by-trial correlation coefficients between the number of spikes during the preselection period (1000–1200 ms after the onset of symbolic cue) and the subsequent saccade metrics across the SEF neurons with direction selectivity (n = 73). Only trials in which saccade was made to the target in the PD of the individual neurons were included in this analysis. Filled bars indicate neurons that exhibited a significant ($p < 0.05$) Pearson correlation. Open bars indicate neurons that did not exhibit a significant correlation. **a** In only 3 neurons (4%), a significant negative correlation between the number of spikes and saccade latency was found. **b** In only 4 neurons (6%, 2 for each of positive and negative correlation), a significant correlation between the number of spikes and saccade peak velocity was found. **c** In only 2 neurons (3%, 1 for each of positive and negative correlation), a significant correlation between the number of spikes and saccade size was observed.
