# Supplementary material for: Preselection of potential target spaces based on partial information by the supplementary eye field
Source: Commun Biol. 2024 Oct 4;7:1215. doi: 10.1038/s42003-024-06878-z (PMC11452695; doi:10.1038/s42003-024-06878-z)
Supplement: Supplementary file 3 — Reporting Summary [file 42003_2024_6878_MOESM3_ESM.pdf]

nature portfolio

Corresponding author(s): Osamu Yokoyama

Last updated by author(s): Aug 23, 2024

Reporting Summary

Nature Portfolio wishes to improve the reproducibility of the work that we publish. This form provides structure for consistency and transparency in reporting. For further information on Nature Portfolio policies, see our Editorial Policies and the Editorial Policy Checklist.

Statistics

For all statistical analyses, confirm that the following items are present in the figure legend, table legend, main text, or Methods section.

n/a	Confirmed	
☐	☒	The exact sample size (*n*) for each experimental group/condition, given as a discrete number and unit of measurement
☐	☒	A statement on whether measurements were taken from distinct samples or whether the same sample was measured repeatedly
☐	☒	The statistical test(s) used AND whether they are one- or two-sided<br>*Only common tests should be described solely by name; describe more complex techniques in the Methods section.*
☐	☒	A description of all covariates tested
☐	☒	A description of any assumptions or corrections, such as tests of normality and adjustment for multiple comparisons
☐	☒	A full description of the statistical parameters including central tendency (e.g. means) or other basic estimates (e.g. regression coefficient) AND variation (e.g. standard deviation) or associated estimates of uncertainty (e.g. confidence intervals)
☐	☒	For null hypothesis testing, the test statistic (e.g. *F*, *t*, *r*) with confidence intervals, effect sizes, degrees of freedom and *P* value noted<br>*Give P values as exact values whenever suitable.*
☒	☐	For Bayesian analysis, information on the choice of priors and Markov chain Monte Carlo settings
☒	☐	For hierarchical and complex designs, identification of the appropriate level for tests and full reporting of outcomes
☐	☒	Estimates of effect sizes (e.g. Cohen's *d*, Pearson's *r*), indicating how they were calculated

Our web collection on statistics for biologists contains articles on many of the points above.

Software and code

Policy information about availability of computer code

Data collection	OpenEX (Tucker-Davis Technologies, Alachua, FL) was used to acquire all data.
Data analysis	OpenSorter (Tucker-Davis Technologies, Alachua, FL, USA) was used to perform spike sorting. MATLAB R2013a Statistics and Machine Learning Toolbox (MathWorks, Natick, MA, USA) was used for statistical analysis and data visualization. CircStat (https://doi.org/10.18637/jss.v031.i10) was used to perform statistical analyses on the circular data. The codes that generate the figures in this paper are available on Zenodo under the identifier: https://doi.org/10.5281/zenodo.13282931.

For manuscripts utilizing custom algorithms or software that are central to the research but not yet described in published literature, software must be made available to editors and reviewers. We strongly encourage code deposition in a community repository (e.g. GitHub). See the Nature Portfolio guidelines for submitting code & software for further information.

Data

Policy information about availability of data

All manuscripts must include a data availability statement. This statement should provide the following information, where applicable:

- Accession codes, unique identifiers, or web links for publicly available datasets
- A description of any restrictions on data availability
- For clinical datasets or third party data, please ensure that the statement adheres to our policy

The behavioral and neuronal data supporting the results reported in this paper are available on Zenodo under the identifier: https://doi.org/10.5281/zenodo.13282931.

Human research participants

Policy information about studies involving human research participants and Sex and Gender in Research.

Reporting on sex and gender	This study did not include human data.
Population characteristics	This study did not include human data.
Recruitment	This study did not include human data.
Ethics oversight	This study did not include human data.

Note that full information on the approval of the study protocol must also be provided in the manuscript.

Field-specific reporting

Please select the one below that is the best fit for your research. If you are not sure, read the appropriate sections before making your selection.

☒ Life sciences ☐ Behavioural & social sciences ☐ Ecological, evolutionary & environmental sciences

For a reference copy of the document with all sections, see nature.com/documents/nr-reporting-summary-flat.pdf

Life sciences study design

All studies must disclose on these points even when the disclosure is negative.

Sample size	No statistical methods were used to predetermine sample sizes, such as the number of animals, neurons, and trials, but they are comparable to previously published neurophysiological studies in nonhuman primates.
Data exclusions	Drifting neuronal activity was excluded from all analyses.
Replication	Qualitatively similar data were obtained from the two monkeys used in each of the experiments in this study.
Randomization	No randomization was performed because the animals were not allocated into groups.
Blinding	Blinding was not relevant to the present study because the animals were not allocated into groups.

Reporting for specific materials, systems and methods

We require information from authors about some types of materials, experimental systems and methods used in many studies. Here, indicate whether each material, system or method listed is relevant to your study. If you are not sure if a list item applies to your research, read the appropriate section before selecting a response.

Materials & experimental systems

n/a	Involved in the study
☒	☐ Antibodies
☒	☐ Eukaryotic cell lines
☒	☐ Palaeontology and archaeology
☐	☒ Animals and other organisms
☒	☐ Clinical data
☒	☐ Dual use research of concern

Methods

n/a	Involved in the study
☒	☐ ChIP-seq
☒	☐ Flow cytometry
☒	☐ MRI-based neuroimaging

Animals and other research organisms

Policy information about studies involving animals; ARRIVE guidelines recommended for reporting animal research, and Sex and Gender in Research

Laboratory animals	This study used 3 Japanese macaque monkeys (Macaca fuscata), monkeys A (female, 5.4 kg, 5 years old at the beginning of the experiment), P (male, 5.6 kg, 4 years old), and K (female, 5.6 kg, 5 years old).
Wild animals	This study did not include wild animals.
Reporting on sex	As noted above.
Field-collected samples	This study did not include field-collected samples.
Ethics oversight	The animals were cared for according to the National Institutes of Health guidelines and the Guidelines of the Tokyo Metropolitan Institute of Medical Science. All animal care and experimental procedures were approved by the Animal Care and Use Committee of the Tokyo Metropolitan Institute of Medical Science (approval nos. 13065, 14070, and 19049). We have complied with all relevant ethical regulations for animal use.

Note that full information on the approval of the study protocol must also be provided in the manuscript.
